# Infectious Diseases—Recent Additions to Our Knowledge

**Published:** 1914-08

**Authors:** 


**Infectious Diseases—Recent Additions to Our Knowledge.** John F. Anderson, U. S. P. H. S., Reprint No. 179 from Public Health Reports, April 3, 1914. Poliomyelitis: filterable virus, germ unknown, communicable to monkeys and occasionally to rabbits, present in emulsion of spinal cord, washings from mouth, nose, trachea, small intestine, transmissible by stable fly, perhaps by other insects, hence not requiring assumption of human carriers that escape infection themselves.

Measles: Almost world-wide in distribution, aggregate mortality slightly higher than for scarlet fever and whooping cough, second only to diphtheria and croup of this class of infections. Filterable virus, germ unknown, communicable to monkeys, but infectivity of blood practically limited to 36 hours after first eruption and of nasal and buccal secretions to 48 hours. Infectivity of dermal scales not established.

Scarlet fever: Filterable virus, transmissible to monkeys, blood emulsion of lymph glands, pericardial fluid, scrapings from tongue infectious; streptococcus apparently excluded, germ unknown.

Typhoid: Bacillus of Eberth shown to be the specific germ, communicable to monkeys but faeces more active than pure cultures. Berkefeld filtrate does not infect nor communicate immunity, hence no filterable virus.

Whooping cough: Bordet-Gengou minute bacillus confirmed, infectious to various young animals, bacilli in masses between cilia of trachea and bronchi, acting mechanically.

Typhus: Identical with Brill's disease, the latter explaining the apparent occurrence of isolated cases of typhus widely separated in time or place from epidemics; semelincident; transmissible to monkeys and other animals; virus in blood; transmissible through lice but not bed bugs; not through buccal and pharyngeal secretions.

